# Supplementary material for: Relationship between the Intake of n-3 Polyunsaturated Fatty Acids and Depressive Symptoms in Elderly Japanese People: Differences According to Sex and Weight Status
Source: Nutrients. 2019 Apr 3;11(4):775. doi: 10.3390/nu11040775 (PMC6521011; doi:10.3390/nu11040775)
Supplement: Supplementary file 1 [file nutrients-11-00775-s001.pdf]

Supplementary Table. Dietary sources of the n-3 PUFA intake.

Dietary sources	%
Grain	1.79
Potato	0.06
Pulse	11.1
Green and yellow vegetables	0.50
Other vegetables	1.44
Fruits	0.16
Fish	49.7
Meats	1.63
Eggs	2.13
Milk	0.38
Oil	19.1
Sweets	1.31
Seasoning/Spices	10.7
